# Trends and patterns in the use of opioids among metastatic breast cancer patients

**DOI:** 10.1038/s41598-020-78569-8

**Published:** 2020-12-10

**Authors:** Chan Shen, J. Douglas Thornton, Kristina Newport, Eric Schaefer, Shouhao Zhou, Nelson S. Yee, Daleela Dodge, Douglas Leslie

**Affiliations:** 1 Department of Surgery, The Pennsylvania State University, College of Medicine, 500 University Drive, H151, Hershey, PA 17033-0850 USA; 2 Department of Public Health Sciences, The Pennsylvania State University, College of Medicine, Hershey, PA USA; 3 Department of Pharmaceutical Health Outcomes and Policy, College of Pharmacy, University of Houston, Houston, TX USA; 4 Department of Medicine, College of Medicine, The Pennsylvania State University, Hershey, PA USA; 5 Penn State Health Milton S. Hershey Medical Center, Hershey, PA USA

**Keywords:** Cancer, Oncology

## Abstract

Opioid use among metastatic breast cancer (MBC) patients has not been well-studied. This study examined the trends and patterns of opioid use among working-age, privately insured patients diagnosed with MBC. Using MarketScan data, we identified female patients diagnosed with MBC in 2006–2015. We determined the proportion of patients who filled a prescription for an opioid and calculated days’ supply and daily morphine milligram equivalents (MMEs) from 1 year prior to diagnosis till 1 year after. We assessed the trend in opioid use over the 10-year study period and examined opioid usage patterns after the diagnosis of MBC. Among 24,752 patients included, 11,579 (46.8%) had an opioid prescription within 1 year before diagnosis of MBC, and 20,416 (81.4%) had an opioid prescription within 1 year after diagnosis. The proportion of patients with opioid prescriptions after diagnosis was relatively stable from 2006 to 2015. However, both the median daily MME and median days’ supply decreased over time with most of the decline from the subgroup of patients with prior prescription opioid use. Most patients received an opioid prescription in the first month after diagnosis (57.3%), dropping to approximately 20% from 3 to 12 months after diagnosis. Also, the median days’ supply increased substantially during the year after diagnosis for patients who received opioids (from 7 to 19). Most women with MBC require opioid analgesia within the first month after diagnosis. Judicious, long-term management of pain after diagnosis of MBC will continue to be necessary for many patients.

## Introduction

Opioid medications are recommended for the management of severe pain in patients with cancer^1^. Prevalence of severe pain is high in patients with cancer, with an estimated 5.39 million people in the United States having cancer‐associated pain with high prevalence (66–72%) in patients with advanced disease^2,3^. Cancer-related pain has significant impact on patients, with 69% of patients reporting pain interfering with activities of daily living^2^. Female patients with advanced cancer are more likely to require opioids for greater than 90 days^4^.

Balancing the benefits of analgesia with the risks of opioid side effects or misuse can complicate prescribing decisions. According to the National Survey on Drug Use and Health (NSDUH), in 2018, 10 million US citizens 12 years of age and older misused prescription opioids and 1.7 million had an opioid use disorder related to prescription opioids^5^. Deaths related to opioid use disorder have garnered significant attention to prescribing practices and have led to more attention to safe and appropriate use of opioids from licensing boards, federal agencies, and the media. These factors have affected prescribing patterns of oncologists with their morphine milligram equivalents (MME) decreasing in one study from 78 in 2010 to 40 in 2015^6^.

Evaluation of opioid use in specific cancer populations is needed to better clarify current use on a cancer-specific basis. Prior studies have evaluated opioid use related to breast cancer surgeries^7,8^, but prescription opioid use in patients with metastatic breast cancer has not been evaluated. With longer survival being the norm for patients with metastatic breast cancer, the need for evaluating their long-term well-being and pain needs is critical. Therefore, the objective of this study was to examine the trends and patterns of opioid use among working-age, privately insured patients diagnosed with metastatic breast cancer. We hypothesize a declining trend in the use of opioids during our study period as clinicians became more aware of the potential negative impacts of opioids. We also hypothesize that some subgroups of patients would still have opioid use over a relatively longer period of time.

## Materials and methods

### Data source

The IBM Truven Health MarketScan database is a de-identified claims-based longitudinal database covering 50 million unique patients enrolled in various commercial health insurance plans including health maintenance organizations (HMOs), preferred provider organizations (PPOs), point-of-service (POS) plans, and indemnity plans and a variety of coverage, such as privately insured fee-for-service, POS, or capitated health plans. The database includes detailed information on demographics, enrollment status in insurance plans, inpatient and outpatient healthcare usage, and prescription drug utilization. It is a well-accepted data source for health utilization and outcomes research^9–12^. We used the MarketScan data up to December 2015 as it was the most recent data available at the time of the study.

### Study cohort

We identified female patients 18–63 years of age, diagnosed with metastatic breast cancer from January 2006 to December 2015 based on a validated claims-based algorithm^13^. Briefly, we used *International Classification of Diseases, Ninth and Tenth Revisions, Clinical Modification* (ICD-9 and ICD-10, respectively) diagnosis codes of 174.x (ICD-9) and C50.x (ICD-10) to identify breast cancer diagnoses. Patients with one inpatient claim or 2 outpatient claims > 30 days apart were classified as having breast cancer. Metastatic breast cancer was identified using ICD-9 diagnosis codes of 196.x to 198.x (excluding 198.2) and ICD-10 diagnosis codes of C77.x to C79.x (excluding C79.2). We classified patients as having metastatic breast cancer if at least 2 claims were found with these codes from 30 days before to any date after the breast cancer diagnosis, with the first claim considered to indicate the metastatic diagnosis date. Patients included in the study were required to have continuous insurance coverage with prescription drugs coverage included in the insurance plan from 12 months prior to and 12 months after the diagnosis date so as to ensure complete records for the identification of pre-diagnosis and post-diagnosis prescription opioid use. The detailed inclusion and exclusion criteria are provided in the Supplementary Figure.

### Data analyses

We identified adjudicated claims for filled opioid prescriptions based on the therapeutic class codes and generic names in the pharmacy claims data. Pharmacy claims with a therapeutic class code of “60: analgesic/antipyretic, opiate agonists” or a generic name including “tramadol” were considered opioid prescriptions; buprenorphine was not included; we excluded a small number of claims that were primarily prescribed as antitussives or Parkinson’s disease such as codeine phosphate/guaifenesin and apomorphine. We calculated the MMEs based on the algorithm by Centers for Disease Control and Prevention (CDC)^14^. In terms of patient characteristics, we calculated the modified Charlson comorbidity index^15^ and also measured whether patients had clinically diagnosed anxiety, depression, severe mental illness and substance use disorder using claims during the 12 months prior to the metastatic diagnosis date based on ICD-9 and ICD-10 codes.

The goal of statistical analysis was to characterize opioid prescription patterns before and after the metastatic diagnosis date. We first analyzed patterns by year, and then analyzed monthly patterns within the year following the metastatic diagnosis date. For both sets of analyses, we also stratified analyses after the metastatic diagnosis date by receipt of an opioid prescription prior to the metastatic index date. For a given time frame (yearly or monthly) we focused on the following three outcomes: (1) proportion of patients with at least one prescription, (2) daily MME, and (3) cumulative days’ supply in the time frame. For the latter 2 outcomes, we reported medians and interquartile ranges (IQRs) due to the skewed distributions of these variables.

For the time trend analysis by year, logistic regression was used to test for trends over time in the proportion of patients who had at least one prescription. Quantile regression^16^ was used to test for differences in median daily MMEs and days’ supply. In all models, year was included as a linear effect, which was appropriate based on regression diagnostics and other graphical analyses. For the analysis by month after the metastatic diagnosis date, we used logistic regression estimated via generalized estimating equations (GEEs)^17^ to analyze the proportion with an opioid prescription. By using GEE, the model accounts for the repeated observations by month after the metastatic diagnosis date. For median daily MMEs and median days’ supply, quantile regression methods appropriate for repeated measures (RQPD)^18^ were used to test medians for significant monthly trends. We included month after diagnosis as a linear effect, with the exception of the proportion of patients who received an opioid prescription, which was highly non-linear. In that case, a b-spline with 3 degrees of freedom was used to test the monthly trend.

The statistical analyses were conducted in SAS 9.4 (SAS Institute, Cary, NC) and R 3.6.0 (R Core Team, Vienna, Austria). The Institutional Review Board at Penn State College of Medicine exempted this study from review because all patients in the database had been de-identified and the study involves no more than minimal risk. No consent process was required since this was deemed exempt from the Institutional Review Board at Penn State College of Medicine. The study was performed in accordance with the ethical standards as laid down in the 1964 Declaration of Helsinki and its later amendments or comparable ethical standards.

### Ethical approval

This study uses de-identified retrospective data and the study was granted exempt status by the Penn State College of Medicine institutional review board.

## Results

A total of 24,752 female patients with metastatic breast cancer diagnosed from 2006–2015 were included in the study. Table 1 provides descriptive characteristics of the study cohort for the date of metastatic diagnosis. The median age was 53 years old with an interquartile range (IQR) of 47–58 years old. Most patients had a comorbidity index of 0 (79.8%). The prevalence of depression and anxiety were both less than 10%, while 4.1% had substance use disorder, and 1.4% had severe mental illness.

**Table 1:** Patient characteristics and opioid prescriptions.

Characteristic	Cohort (N = 24,752)
Age in years, median (IQR)	53 (47–58)
**Year of metastatic diagnosis date, N (%)**	
2006	1505 (6.1%)
2007	2014 (8.1%)
2008	2222 (9.0%)
2009	2521 (10.2%)
2010	2601 (10.5%)
2011	3102 (12.5%)
2012	2621 (10.6%)
2013	2626 (10.6%)
2014	2673 (10.8%)
2015	2867 (11.6%)
**Region, N (%)**	
Northeast	3736 (15.1%)
North central	5951 (24.0%)
South	10,188 (41.2%)
West	4712 (19.0%)
Unknown	165 (0.7%)
**Charlson comorbidity index, N (%)**	
0	19,750 (79.8%)
≥ 1	5002 (20.2%)
Anxiety, N (%)	2116 (8.5%)
Depression, N (%)	2202 (8.9%)
Severe mental illness, N (%)	351 (1.4%)
Substance use disorder, N (%)	1011 (4.1%)
Opioid prescription within 1 year prior to metastatic diagnosis date, N (%)	11,579 (46.8%)
**Number of opioid prescriptions [among patients with use prior to diagnosis], N (%)**	
1	5616 (48.5%)
2	2327 (20.1%)
3	1086 (9.4%)
4	594 (5.1%)
5	352 (3.0%)
6	295 (2.5%)
≥ 7	1309 (11.3%)
Opioid prescription within 1 year after metastatic diagnosis date, N (%)	20,146 (81.4%)
**Number of opioid prescriptions [among patients with use after diagnosis], N (%)**	
1	4903 (24.0%)
2	4127 (20.2%)
3	2701 (13.2%)
4	1833 (9.0%)
5	1279 (6.3%)
6	913 (4.5%)
≥ 7	4660 (22.8%)

Summary statistics regarding opioid prescriptions for the cohort are displayed in Table 1. A total of 11,579 patients (46.8%) had an opioid prescription during the 1 year prior to the metastatic breast cancer diagnosis date. Among these patients, the majority (51.5%) had at least two opioid prescription claims, 31.4% had at least three opioid prescription claims, and 11.3% had at least seven filled opioid prescriptions. During the 1 year after metastatic breast cancer diagnosis, 20,416 patients, a larger percentage (81.4%), received at least one opioid prescription. Among these patients, the majority (55.8%) had at least three opioid prescription claims, and 22.8% had at least seven filled opioid prescriptions.

**Figure 1:**
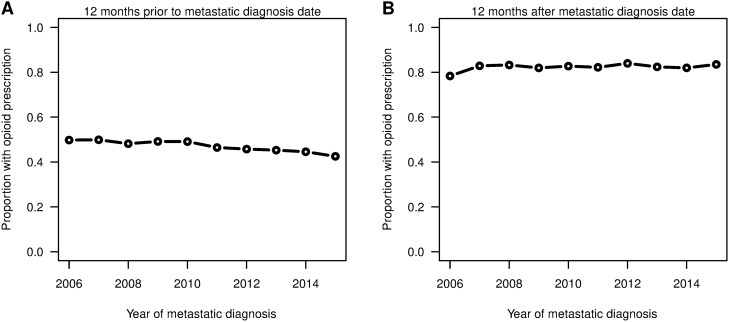
Proportion of patients who filled a prescription for an opioid within 12 months before the metastatic diagnosis date (left) and 12 months after the metastatic diagnosis date (right). Using R software (https://www.R-project.org/).

**Figure 2:**
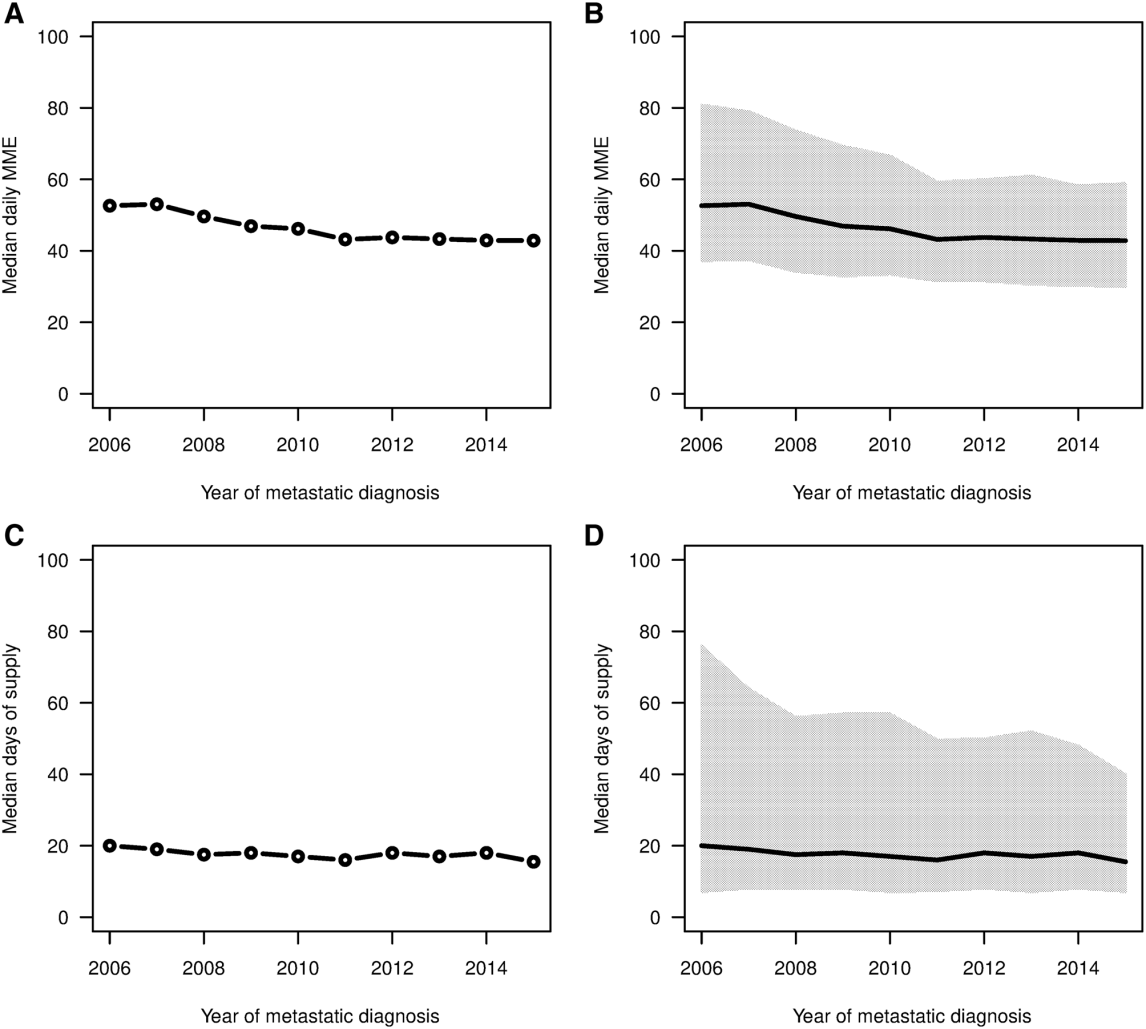
Median daily MMEs and median days of supply within 12 months of the metastatic diagnosis date (left) with IQRs (right) by calendar year for patients who had at least one opioid prescription after the metastatic diagnosis date. Using R software (https://www.R-project.org/).

Figure 1 shows the proportion of patients who filled opioid prescriptions 12 months before and after metastatic breast cancer index date. For the 2006 to 2015 study period, the proportion of patients with opioid prescriptions 1 year prior to the metastatic diagnosis date showed a significant decreasing trend (P < 0.001) from 49.8% in 2006 to 42.5% in 2015. For the year after the metastatic diagnosis date, the proportion slightly increased (P = 0.028) from 78.3% in 2006 to 83.5% in 2015. Figure 2 shows the median daily MME and median days’ supply for opioids with IQRs after the metastatic diagnosis date among patients who had at least one opioid prescription. Both the median daily MME and median days’ supply decreased significantly (both P < 0.001) for the years observed. Further, we found that IQRs shrank, especially for the upper quartiles. The upper quartile of daily MME decreased from 81 to 59, and the upper quartile of days’ supply decreased from 76 to 40 over the time period.

**Figure 3:**
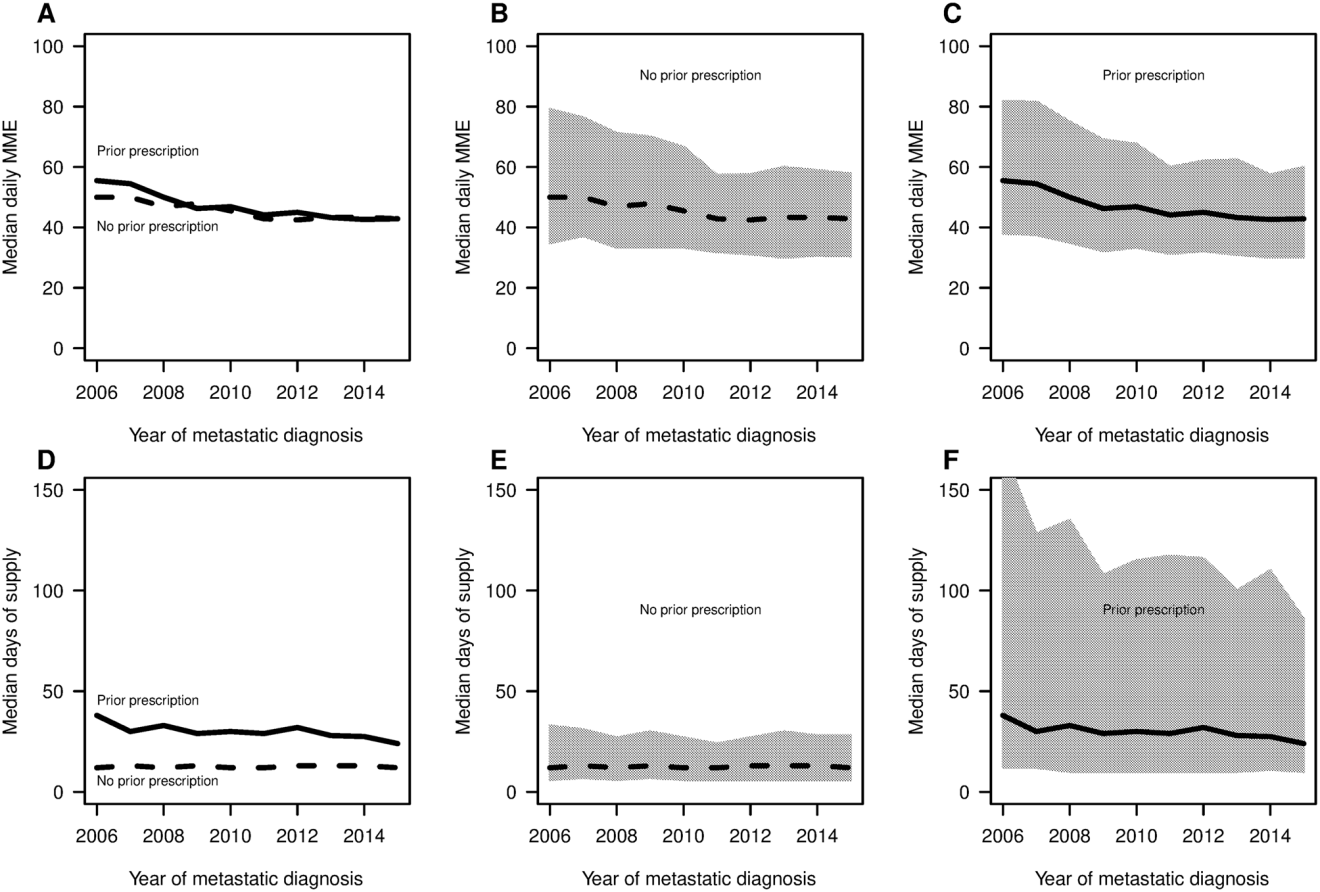
Median daily MMEs and median days of supply within 12 months of the metastatic diagnosis date by year stratified by use prior to the date (leftmost figures). The rightmost panels show the respective IQRs. Using R software (https://www.R-project.org/).

In Fig. 3, we compared patients with and without prior opioid use in terms of daily MME and days’ supply. Patients with prior use had both higher median daily MME and larger median number of days’ supply. Patients with and without prior use both had decreased median daily MME (P < 0.001) over time. More specifically, patients with prior use had median daily MMEs decrease from 55.5 to 42.9, while patients without prior use a decrease from 50.0 to 42.9. The results between the two subgroups were more striking for median days’ supply, which decreased significantly (P < 0.001) for patients with prior use from 38 to 24, but was stable (P = 0.07) for patients without prior use at a value near 12. Comparing the IQRs, we observe 7.5 times larger range in 2006 for patient with prior use (IQR of 12–170) compared to patients without prior use (IQR of 12–33). However, the upper quartile for days’ supply for patients with prior use decreased dramatically from 170 to 86 over the study period.

**Figure 4:**
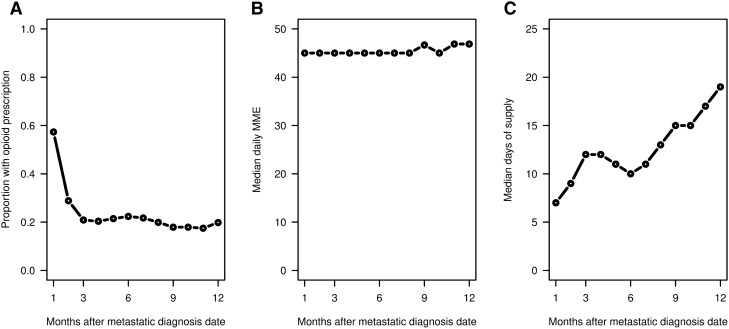
(Left) Proportion of patients with a prescription for an opioid by month after the metastatic diagnosis date. (Middle) Median daily MMEs and (Right) Median days of supply for patients who had a prescription for an opioid within the month. Using R software (https://www.R-project.org/).

**Figure 5:**
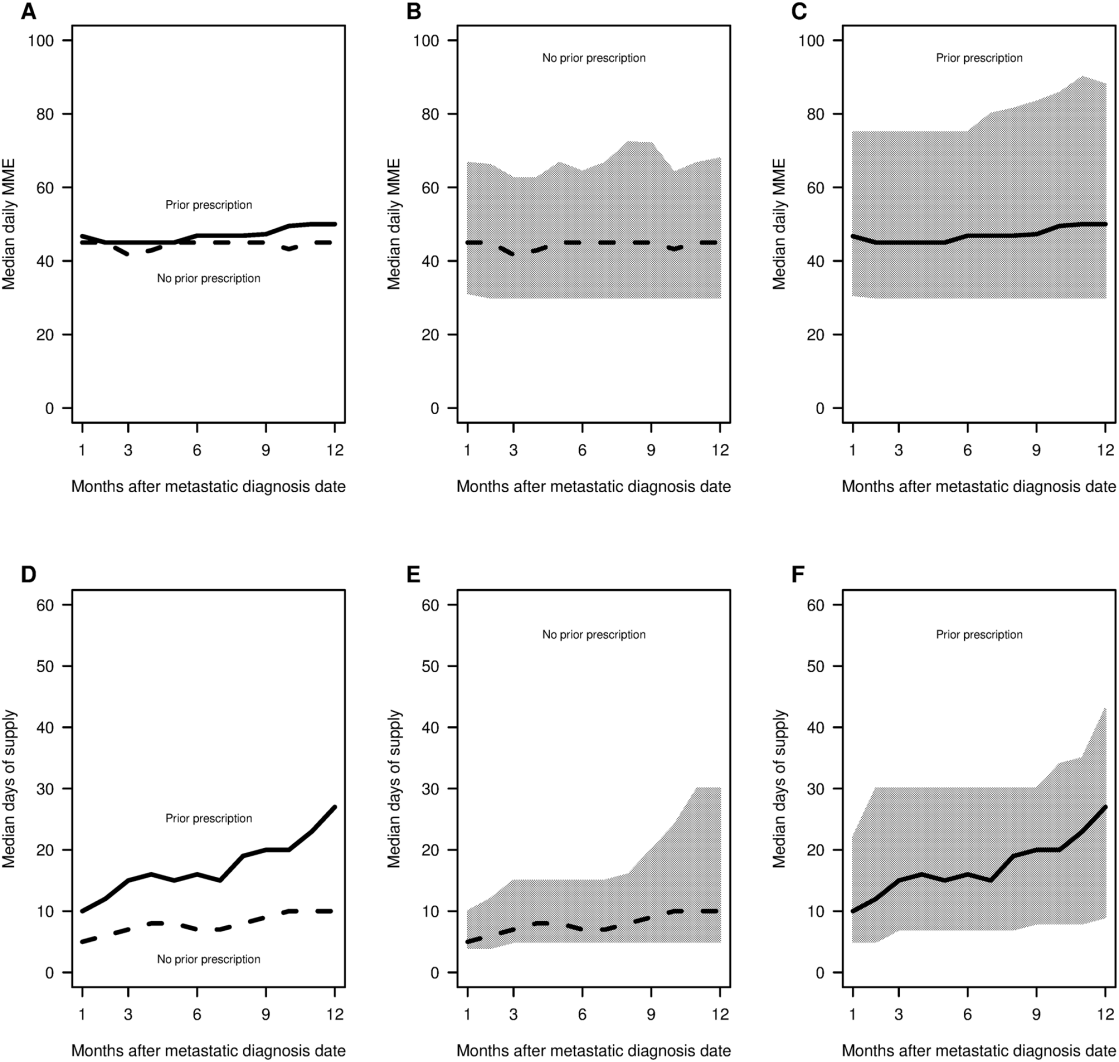
Median daily MME and median days of supply by month (left panel) after the metastatic diagnosis date stratified by any previous opioid use among patients who filled a prescription in the month. The rightmost panels show the respective IQRs. Using R software (https://www.R-project.org/).

Figures 4 and 5 focus on the monthly pattern of opioid use for patients during the 12 months after metastatic breast cancer diagnosis. Figure 4 provides the proportion of patients with opioid prescriptions by month, and the median daily MMEs and median days’ supply by month among patients who received an opioid prescription in those months. The proportion of patients receiving an opioid prescription was highest in the first month at 57.3%, then sharply decreased to 28.3% in the second month, and then stabilized to approximately 20% from the third to twelfth months. The median daily MME was stable at 30 during the 12 months after diagnosis for patients who received an opioid prescription. However, the median days’ supply among patients who received an opioid prescription rose significantly (P < 0.001) from 7 to 19 by month after metastatic diagnosis date. Figure 5 shows monthly opioid prescription patterns for patients with and without prior use. Both subgroups of patients had increasing median days’ supply over the 12 months (P < 0.001). Patients without prior use had increased median days’ supply from 5 in the first month to 10 in the 12th month, compared to 10–27 for patients with prior use. For patients with prior use, IQRs of days’ supply were also 2–3 times larger.

## Discussion

To the best of our knowledge, this is the first large, observational study focusing on the opioid use time trend and patterns among working-age patients with metastatic breast cancer. We found that nearly half of women diagnosed with metastatic breast cancer received opioid prescriptions prior to their diagnosis, but this proportion significantly decreased over time from 49.8% in 2006 to 42.5% in 2015, possibly reflecting the scrutiny of opioid prescribing. It is likely that many of these patients with prior opioid use had a diagnosis of breast cancer earlier. In our study cohort, close to half (46.9%) of the patients received their metastatic diagnosis within 30 days from the first breast cancer diagnosis date, while the rest of the patients had their metastatic diagnosis date more than 30 days after the first breast cancer diagnosis. Prior studies have demonstrated past opioid use as predictive of future opioid use, consistent with our findings of higher doses for longer time in patients with opioid use prior to metastatic diagnosis^4^. Clinicians need to be cognizant of the pain treatment regimens and heterogeneous needs of the patients during their cancer treatment.

After diagnosis, a large proportion of this study sample (> 80%) received at least one opioid prescription indicative of a uniform and consistent need for pain management. Although receipt of at least one opioid prescription remained high over time, there was a substantial decrease in both daily MME and days’ supply. During the course of the study from 2006 to 2015, there was an overall reduction in opioid prescribing. We observed much reduced practice variation in opioid prescribing (IQR range much smaller), especially in the upper quartile. This reduction in dosage may be driven by changing prescribing practices with clinicians becoming more aware of the negative impacts of potential opioid misuse.

External factors are also associated with patients filling opioid prescriptions including prior authorization requirements, community pharmacy limits, and an increased emphasis on the potential risks of opioid medications. Other studies have examined the trends in opioid use and prescribing in the general population have often found no significant reduction in opioid use and showed heterogeneous patterns. For example, one study focusing on the initiation of opioid prescription among privately insured patients found declines in the monthly incidence of initial opioid prescriptions 2002–2017; however, a subgroup of providers continued to prescribe high-risk opioid therapy^19^. A study examining opioid use in commercially insured and Medicare Advantage beneficiaries also showed no substantial decline in opioid use and average daily dose from 2007 to 2016^20^. Another study found stable opioid use overall among Medicare patients from 2006 to 2012 with increasing variations in the intensity of use, patient diagnoses and prescriber specialty^21^. Therefore, it seems that our study focusing on metastatic breast cancer patients showed significantly more reduction in opioid use and also notably decreasing variations compared to the more general population. It highlights the importance of differentiating subgroups of patients with specific conditions when studying the use of opioids.

The variation in opioid prescribing was substantially larger in patients with prior prescriptions. This contributed to most of the reduction in practice variation being observed in patients who had prior opioid use over the study period. The upper quartile for days’ supply reduced tremendously by about a half from 170 to 86 for patients with prior use, showing much more scrutiny in opioid usage among this group of patients. It is worth noting that the median daily MME decreased to 42.9 for both patients with and without prior opioid use in 2015, although the numbers were 55.5 and 50.0 for patients with and without prior use respectively in 2006. It seems that clinicians were increasingly aware of the potential harm of opioids, which led to reduced daily MME below 50. Although there is no widely accepted guideline for opioid prescription among metastatic breast cancer patients, there are many studies in the literature on other populations that suggested that daily dosages above 50 MME is associated with higher risk for opioid overuse, which led the Centers for Disease Control to recommend clinicians carefully reassess evidence of individual benefits and risks when considering increasing dose to > 50MME per day and avoid prescribing > 90MME per day in 2016^22^. Although this did not apply specifically to people with cancer, this opioid threshold was adopted by some regulatory organizations and insurance companies, therefore potentially impacting the prescribing habits of clinicians^22^.

In terms of monthly use patterns after diagnosis, a large proportion of the patients stopped using opioids during the first 2 months, presumably correlating with pain caused by cancer that was improved with treatment. The days’ supply increased significantly among the patients who continued to take opioids over the 12 months. These findings suggest that there is a subgroup of advanced breast cancer patients who may have persistent pain requiring longer term treatment with opioids. Metastatic breast cancer patients can have pain due to metastases to various organs such as bone, central nervous system, gastrointestinal tract, pulmonary system, and lymph nodes. Again, chronic pain is highly prevalent among advanced cancer patients ranging from 62 to 90%^23,24^. The pain could be neuropathic pain or nociceptive pain, so a well-managed pain management regimen, possibly including opioids, may be beneficial for these patients.

Survival for metastatic breast cancer patients has been improving steadily. One study documented improved median survival from 23 to 29 months and 5 year survival rates from 11 to 28% between 1987–1993 and 1994–2000^25^. Another more recent study examining survival trend of metastatic breast cancer from 1990 to 2011 also showed an improvement in overall survival over time and a median overall survival of 28 months by 2008–2011^26^. Chemotherapy, anti-growth factor antibodies, tyrosine kinase inhibitors, and endocrine therapy are standard treatments for metastatic breast cancer^27^. We expect the survival of metastatic breast cancer patients to continue to improve with advancements in the treatment for metastatic breast cancer. In recent years, new therapies such as pertuzumab^28^, ado-trastuzumab emtansine^29^, ribobiclib^30^, abemaciclib^31^, atezolizumab^32^ have been approved and are favorably impacting survival. More women will be living longer with metastatic cancer, so appropriate pain management strategies are critical.

This is an observational study with the common limitations due to the nature of claims data analyses. For example, the database does not include detailed information about the tumor characteristics such as tumor size, histology, gene mutations, etc. This study is based on claims data on working-age patients enrolled in commercial health insurance plans, and therefore does not include patients under Medicare and Medicaid, who might differ in their rate of metastatic breast cancer diagnosis and frequency of opioid prescribing. Also due to the claims-based nature of the data, we do not have longer term survival follow-up information of the patients. We did require that patients included in the study had at least 1 year of continuous enrollment in their insurance plan therefore they survived at least 1 year after their metastatic breast cancer diagnosis.

This study did not assess the level of pain for which opioids were prescribed due to the limitation of claims data analyses. We therefore are unable to determine whether the decrease in dose and duration of opioid prescribing correlated with appropriate analgesia or resulted in unmet needs. The potential unmet needs for pain management due to less use of opioids is certainly a substantial concern for this population. Future research is warranted to study whether and what type of other alternative pain management strategies are used and whether patients receive appropriate pain management.

## Conclusions

This large observational study showed most women with breast cancer require opioid analgesia within the first month after diagnosis of metastatic breast cancer and a large proportion of them received an opioid prescription before metastatic diagnosis. There was a decreasing trend in the daily MME and days’ supply of opioid prescriptions over the study period. Many patients filled opioid prescriptions only in the first two months immediately after metastatic diagnosis. However, the subgroup of patients who had more persistent opioid use (> 90 days) had increasing opioid prescriptions potentially indicating ongoing cancer-related pain.

## Supplementary Information

Supplementary Figure.

## Data Availability

The datasets generated during and/or analysed during the current study are available from the corresponding author on reasonable request.
